# Depressive symptoms as a cause and effect of job loss in men and women: evidence in the context of organisational downsizing from the Swedish Longitudinal Occupational Survey of Health

**DOI:** 10.1186/s12889-015-2377-y

**Published:** 2015-10-12

**Authors:** Elena Andreeva, Linda L. Magnusson Hanson, Hugo Westerlund, Töres Theorell, M. Harvey Brenner

**Affiliations:** FG Epidemiologie, Fakultät VII, Technische Universität Berlin, Berlin, Germany; Centre for Applied Rehabilitation Research, Department of Rehabilitation Medicine, Hannover Medical School, Hannover, Germany; Stress Research Institute, Stockholm University, Stockholm, Sweden; Department of Behavioral and Community Health, School of Public Health, University of North Texas Health Science Center, Fort Worth, TX USA; Johns Hopkins University Bloomberg School of Public Health, Baltimore, MD USA

**Keywords:** Depression, Organisational downsizing, Job loss, Employment status, Sweden

## Abstract

**Background:**

Few studies have examined depression as both a cause and effect of unemployment, but no prior work investigated these relationships in the context of organisational downsizing. We explored whether the exposure to downsizing is associated with subsequent depression (social causation), and whether pre-existing depression increases the risk of being laid off when organisations downsize (health selection).

**Methods:**

Two successive waves of the nationally representative Swedish Longitudinal Occupational Survey of Health represented the baseline (2008) and follow-up (2010) of this study. Analyses included 196 workers who lost their jobs through downsizing, 1462 layoff survivors remaining in downsized organisations and 1845 employees of non-downsized workplaces. The main outcomes were: (1) Depressive symptoms at follow-up, assessed with a brief subscale from the Symptom Checklist 90, categorised by severity levels (“major depression”, “less severe symptoms” and “no depression”) and analysed in relation to earlier downsizing exposure; (2) Job loss in persons with downsizing in relation to earlier depressive symptoms. The associations were assessed by means of multinomial logistic regression.

**Results:**

Job loss consistently predicted subsequent major depression among men and women, with a somewhat greater effect size in men. Surviving a layoff was significantly associated with subsequent major depression in women but not in men. Women with major depression have increased risks of exclusion from employment when organisations downsize, whereas job loss in men was not significantly influenced by their health.

**Conclusions:**

The evidence from this study suggests that the relative importance of social causation and health selection varies by gender in the context of organisational downsizing. Strategies for handling depression among employees should be sensitive to gender-specific risks during layoffs. Policies preventing social exclusion can be important for female workers at higher risk of depression.

## Background

Since the early 1970s, and increasingly after the 1990s, there has been a trend in business practice toward frequent workforce downsizing. This trend has become intense during the Great Recession which began in Sweden in late 2008. While downsizing can be important for survival or competitiveness of economic organisations, it is often associated with depressive symptoms among laid off [1, 2] and remaining [3–5] workers. In the attempt to explain these associations, previous research considered the processes of social causation and health selection. The social causation perspective emphasizes the importance of psychosocial stressors in the development of mental illness, whereas health selection theory argues that illness leads to a decline or downward drift in social status. Evidence from prospective studies suggests that major downsizing has the potential to harm the emotional health of workers through job insecurity [6, 7], job displacement [1, 2], unemployment [8, 9] or surviving substantial layoffs while staying employed in downsized companies [10, 11] (i.e., social causation), although these findings are not entirely consistent [12, 13]. Less frequently, longitudinal investigations inferred reverse causality, with the suggestion that mental ill health and depression in particular increases the risk of unemployment (i.e., health selection) [14]. Finally, few prospective studies have analysed depressive affect as both consequence of and risk factor for unemployment and found a bidirectional linkage between these conditions [15–18]; yet none of these studies have focused on downsizing events during economic recessions. Thus, evidence remains limited as to whether such downsizing can (a) cause new or worsening depression in displaced and remaining workers and (b) intensify social exclusion of employees with symptoms of depression through unemployment.

This study focusses on downsizing events with compulsory redundancies, that is, layoffs due to insufficient work being available in one’s place of employment [19]. Downsizing with compulsory redundancies is known to have stronger adverse effects on health than staff reductions achieved without immediate job losses, for example, by means of reduced work hours or voluntary turnover [20]. Layoffs usually occur in response to short-term needs and external events, such as economic decline [21]; they are collective events in which general economic circumstances frequently affect large segments of the organisation’s workforce. Unemployment due to layoffs is in principle less individually selective than job loss caused by personal characteristics of employees [19]. In other words, ill health may not be the critical factor for losing one’s job during layoffs; therefore, the effects of health selection may not become apparent. According to the “last in first out” (LIFO) rule, the worker who has been employed last is most vulnerable to job loss when an organisation downsizes. However, the empirical evidence on the impact of this rule on dismissals does not clearly support the theoretical assumptions [22]. Workers with records of significant illness may be targeted for redundancy more often than their healthy colleagues.

Given the importance of depression for workforce productivity and labour force participation, understanding the nature of relationships between depression symptoms and compulsory redundancies is crucial for informing prevention, treatment and active labour market policies at times of economic downturn. Specifically, this may concern early recognition and treatment of depressive symptoms in employees of downsized organisations, as well as policies aimed at social inclusion of laid-off workers with depression. Using data from two successive waves of the nationally representative Swedish Longitudinal Occupational Survey of Health (SLOSH), we investigated social causation and health selection with two main research questions: (1) whether the exposure to downsizing during the recent Great Recession is associated with subsequent depression in laid-off and remaining workers and (2) whether pre-existing depression increases the risk of being laid off when organisations downsize. While women are known to have a higher prevalence of depression, the relative importance of social causation and health selection has not been sufficiently addressed by gender in the context of organisational downsizing. The aim of this study was therefore to analyse the gender-specific associations and the relationships in both sexes combined.

## Methods

### Study population

For the present study, participants were originally recruited from a large representative sample of the Swedish working population in 2003–2005 arising out of the Swedish Work Environment Survey (SWES). SLOSH was conceived as a follow-up to SWES with a more detailed prospective collection of data on work environment and health [23]. The SLOSH population is surveyed biennially since 2006 by self-administered postal questionnaires. Participants fill in one of the two questionnaires: one addressed to workers gainfully employed for at least 30 % of full time, or one designed for “not gainfully employed” respondents working less than that or not at all; the latter category includes mostly persons outside the labour force (homemakers, non-working students, pensioners etc.). From 2008 onwards, the SLOSH questionnaires make it possible to assess the impact of company downsizing and the resulting changes in employment status on the health of the workers. Therefore, we used data collected in the second (2008) and third (2010) waves of SLOSH. Data collection in 2008 occurred before the outbreak of the Great Recession. In this report 2008 is treated as baseline and 2010 as follow-up.

In total, 8771 persons returned a questionnaire in both 2008 and 2010; attrition rate between these waves was 23.3 %. Those with missing or incomplete data on education or depressive symptoms (*N* = 418) were excluded from the analyses. Given our research focus, we further excluded respondents who were unlikely to experience collective compulsory redundancies during the Great Recession:

- 1179 self-employed, farmers and workers of microenterprises with less than 10 employees;
- 1800 workers of larger enterprises with some downsizing but no collective compulsory redundancies (questions on compulsory redundancies not completed);
- 1529 respondents considered economically inactive by ILO definition [24] (non-working students, homemakers, retirees) or non-employed for reasons other than downsizing during the Great Recession.

This perspective was adopted for the following reasons: (a) Downsizing in microenterprises (including farms) is not subject to the stringent LIFO rule stipulated in the Employment Protection Act [25]: exemptions of “key workers” from this rule may affect both the downsizing procedure [22] and mental health outcomes, possibly resulting in artificial inflation of the results due to a higher level of psychological distress. (b) Staff reductions without compulsory redundancies can represent strategic downsizing aimed at promotion of long-term organisational benefits. This approach may be associated with a less detrimental health impact due to support and employment of surplus workforce [21] (inclusion of persons affected may result in artificial deflation of the results). (c) Being non-employed might be associated with poorer mental health (i.e. artificial deflation of the results due to the inclusion of voluntarily unemployed or disabled persons in the reference group).

Furthermore, given our research focus on people who cannot withdraw from situations of compulsory redundancies – workers who lost their jobs and layoff survivors – we have also excluded 238 employees of downsized organisations who retired, quit or found another job before becoming unemployed. These exposures might be associated with either poorer or better mental health. Finding another job could represent a healthy outcome to impending layoffs, as healthier and better educated workers may find it easier to obtain new employment before the actual job loss. In contrast, older and less healthy workers are overrepresented in jobs that have become obsolete as a result of technological developments [26]. In fact, difficulties obtaining new employment may be influencing the decision to retire early. Small numbers and a highly heterogeneous composition of respondents would prohibit a detailed analysis of relationships in this group.

Finally, because of missing data on either organisation size, permanence of employment, self-employed/farmer status or downsizing exposure, we excluded another 104 persons.

Consequently, the final analytic sample consisted of 3503 persons. The sample comprised three groups of permanent and temporary workers employed in small (10 to 49 workers), medium and large organisations (≥50 workers): (a) 1845 workers in companies with no downsizing, (b) 1462 layoff survivors, who remained at work and (c) 196 displaced workers who lost their jobs through compulsory redundancies.

### Dropout analysis and representativeness of the analytic sample

We conducted a dropout analysis to test, whether dropout between the 2008 and 2010 SLOSH waves was related to demographic characteristics (age, gender, marital status and education), employment and depression. Results from a multivariate logistic regression (outcome: loss to follow-up in 2010 versus responding to SLOSH in both 2008 and 2010) indicate that dropout is significantly predicted by male gender, younger age, lower education, non-permanent employment and being single, all *p* < 0.001. Similar patterns of dropout were earlier reported for non-respondents to SLOSH in relation to SWES participants [27, 28]. Yet our results indicate that depression did not significantly affect the likelihood of loss to follow-up in 2010 (*p* > 0.05).

Furthermore, using descriptive statistics, we checked whether our analytic sample can be regarded as representative for the sample of all respondents to the 2008 SLOSH wave. Compared to the total sample, the analytic sample included higher proportions of men (46 vs. 45 %), singles (45 vs. 44 %), university educated (39 vs. 36 %) and permanently employed workers (95 vs. 88 %) and had a lower mean age (48 vs. 49 years). However, the distribution by the level of depression at baseline showed that the proportions of non-depressed respondents were equal in both samples (76 %). Thus, the representativeness of the analytic sample is adequate with respect to likely depression prevalence.

### Ethics

The study was approved by the Regional Research Ethics Board in Stockholm (Ref.no: Dnr 2006/158-31, Dnr 2008/240-32 and 2010/0145-32). All subjects gave their written informed consent. The entire data collection was carried out by Statistics Sweden on behalf of the Stress Research Institute at Stockholm University. Statistics Sweden delivered the data to the researchers in such a form that neither individuals nor groups based on, e.g., employment at a certain workplace, could be identified.

### Measures

#### Exposure to downsizing during the Great Recession

Downsizing is defined as a process whereby an organisation reduces its personnel, particularly, through redundancy [19]. All participants were asked in 2010 whether they had experienced downsizing in the last two years. The *unexposed group* answered this question in the negative; job losses for other reasons were precluded. Persons with “yes”-responses were further prompted to specify a proportion of the employees made redundant on a scale including “less than 8 %”, “between 8 and 18 %” and “18 % or more”. People with non-missing responses to this item were further classified based on the question: “In what way were you personally affected by the changes?”. Exposed subjects, who answered that they received a warn notice and became unemployed, were classified as *displaced workers*. The exposed group of *layoff survivors* included persons continuously employed in the same downsized organisation, both notified workers who did not have to leave and those never notified. We coded the variable as 1 = employees in non-downsized organisations (reference group), 2 = layoff survivors and 3 = workers displaced due to downsizing.

### Depressive symptoms

Depressive symptoms in 2008 and 2010 were assessed by a brief version of the depression subscale of the Hopkins Symptom Checklist 90 [29]. The scale (SCL-CD_6_) measures one-week prevalence and includes six items covering the core symptoms of depression: lowered mood (“feeling blue”), loss of interest (“feeling no interest in things”), reduced energy and diminished activity (“feeling lethargy or low in energy”), marked tiredness and, possibly, psychomotor retardation (“feeling that everything is an effort”), excessive worries reflecting psychic anxiety, phobic, hypochondriac or obsessional symptoms (“worrying too much about things”), and self-accusation due to feelings of guilt or unworthiness (“blaming yourself for things”). This scale is particularly suitable for assessment in large population surveys because of its brevity, ease of administration and the central role of clinical validity in the selection of items [30]. Respondents rated how much they have been troubled by each symptom on a five-point Likert scale. The total sum score ranges from 0 to 24. The SCL-CD_6_ with a coefficient of homogeneity of 0.70 by Mokken analysis indicates a meaningful dimensional measure of depression severity. The scale has proven to be valid and had higher uni-dimensionality than longer epidemiologic instruments, thus being more specific for measuring depression as the underlying construct. The standardisation of the SCL-CD_6_ was based on receiver operating characteristic analysis, using the Major Depression Inventory as an index of validity. A score of 17 or higher was found to be the best cut-point for major depression (sensitivity 0.68, specificity 0.98), with significantly predicted subsequent use of antidepressants and hospitalisations for depressive episodes [31]. The subjects were classified in accordance with their score values as being likely to have major depression (from 17 to 24), less severe depression symptoms (from 10 to 16) and no depression (from zero to nine) [31, 32]. The variables denoting the level of depression at baseline and follow-up include three categories based on this classification: 1 = no depression, 2 = less severe depression symptoms and 3 = major depression.

### Covariates

*Demographic factors* included age, sex (in the analyses combining both genders) and education, derived from national registers, and self-reported marital status. These factors are regarded as potential confounders as they may influence the experience of unemployment and depression [33, 34]. Education has been the key proxy for socioeconomic status (SES) in earlier studies: it has the advantage of relative stability across the life course in adults. Moreover, education is less prone to bias of reverse causality (i.e. health affecting SES) than measures like income and occupation [35]. We linked the register-based information with the questionnaire data by means of unique ten-digit personal identification numbers. Age was measured in years, educational level included three categories: 1 = mandatory education only, 2 = high school or comparable, 3 = university degree. Marital status was assessed with a direct question and coded as 1 for married/cohabiting and 0 for single.

*Employment variables* included permanence of employment at baseline and a measure of changes in the employment status at follow-up. We controlled for these variables in order to account for a potentially adverse impact of lost seniority after losing a permanent job [22] and to consider the effects of reemployment: finding paid employment is known to reduce depression risks in displaced workers [36]. Permanence of employment was assessed with a direct question and coded as 0 for various types of temporary employment, such as project-based or substitute, and 1 for permanent employment. Regarding employment status, respondents were categorised by the type of questionnaire they completed in 2010 as being either “gainfully employed” for at least 30 % of full time (code 1), or “not gainfully employed” i.e. working less than that or not at all during the past 3 months (code 0).

We also adjusted for *past redundancies* to exclude the possibility that the observed depression risks are due to long-term psychological scarring from earlier layoffs [37] prior to the recession. This variable is coded as 1 if respondents indicated in 2008 that they had survived layoffs or had been laid off in the previous two years (2006–2008); otherwise it is coded as 0.

A self-reported measure of *long-term sickness* captures underlying chronic medical and psychiatric conditions, which may be associated with depressive symptoms and influence one’s experience of unemployment [38, 39]. It is based on the information on long-term leaves with sickness benefits, activity or sickness compensation (0 = no long-term sick leaves in both 2008 and 2010; 1 = long-term sick leaves in 2008 or 2010).

### Statistical analysis

For all statistical analyses, we used the STATA software package, version SE 11.2. First, we calculated descriptive statistics (numbers and percentages, means and standard deviations (SD)) and evaluated the bivariate gender-specific associations of socio-demographic and health characteristics with the exposure status using Pearson’s *χ*2 test and analysis of variance, when appropriate. Significance was considered at *p* < 0.05.

Second, we performed multivariate analyses of relationships in line with our research questions. The first set of multivariate analyses examined job displacement and surviving a layoff during the Great Recession as key predictors of depressive symptoms at follow-up (social causation). Relative risk ratios (RRR) with 95 % confidence intervals (CI) were estimated from multinomial logistic regression models. While risks of depressive disorders are generally higher in women, job displacement may be more detrimental in men due to demands posed by the traditionally male-gendered responsibility for breadwinning [40]. Therefore, in addition to estimating the strength of relationships in the total analytic sample, we performed analyses stratified by gender, while adjusting for demographic and employment variables, depression at baseline, past redundancies and long-term sickness. Exposure status, education and depression at baseline were treated as factor variables: this procedure creates dummy variables for the levels of categorical regressors [41].

In the second set of multivariate analyses, we examined whether pre-existing depression increases the risk of being laid off (i.e. becoming unemployed) when organisations downsize (health-related selection). These analyses were restricted to 1658 victims (i.e. displaced workers) and survivors of layoffs during the Great Recession. Unemployment at follow-up was coded as 1 for displaced workers and 0 for layoff survivors. Level of depression at baseline was treated as the key explanatory factor variable. The multinomial logistic regression models for men, women and both sexes combined were adjusted for variables which can affect the probability of losing a job during downsizing, including demographic factors, permanence of employment, past redundancies, long-term sickness and scale of downsizing during the Great Recession (dichotomously coded: large versus minor staff reductions of less than 8 %).

## Results

### Descriptive statistics and bivariate associations

The analytic sample comprised 1624 (46 %) men and 1879 (54 %) women. Women had a mean age of 49.8 years (SD ± 9.9); men were slightly younger (mean age 49.7 years, SD ± 9.9). Table 1 shows the descriptive statistics of the study participants by gender and exposure status. We found significant and consistent gender-specific relationships between the exposure statuses and education (displaced workers being less well educated), permanent employment at baseline (temporary employment being most frequent among the displaced), gainful employment at follow-up (least common in displaced workers), long-term sickness (more prevalent in those displaced) and prevalence of depression (higher at baseline and follow-up in both downsized groups), all *p* < 0.01. Further results indicate in both genders that past redundancies were most frequent among the victims and survivors of layoffs; a larger scale of downsizing during the Great Recession was more common in displaced workers (all *p* < 0.001). Age was significantly related to the exposure status only in men (displaced workers were older, *p* < 0.05). Relationships with marital status were significant only in women (more laid off respondents being single, *p* < 0.01).

**Table 1:** Characteristics of study participants by gender and exposure to downsizing (N = 3503)

	Men				Women			
Characteristic	Displaced workers (*N* = 112)	Layoff survivors (*N* = 754)	No downsizing (*N* = 758)	*p value	Displaced workers (*N* = 84)	Layoff survivors (*N* = 708)	No downsizing (*N* = 1087)	*p value
Age, years: mean ± SD	50.5 ± 10.4	49.0 ± 9.8	50.3 ± 10.0	0.032	49.2 ± 11.2	49.2 ± 9.9	50.3 ± 9.8	0.074
Education				<0.001				<0.001
Mandatory	19 (17.0)	83 (11.0)	91 (12.0)		17 (20.2)	48 (6.8)	60 (5.5)	
High school	77 (68.7)	460 (61.0)	398 (52.5)		49 (58.3)	333 (47.0)	517 (47.6)	
University	16 (14.3)	211 (28.0)	269 (35.5)		18 (21.4)	327 (46.2)	510 (46.9)	
Marital status				0.088				0.003
Married/cohabiting	56 (50.0)	424 (56.2)	454 (59.9)		33 (39.3)	380 (53.7)	626 (57.6)	
Single	56 (50.0)	330 (43.8)	304 (40.1)		51 (60.7)	328 (46.3)	461 (42.4)	
Permanence of employment at baseline				<0.001				0.002
Permanent	98 (87.5)	718 (95.2)	734 (96.8)		72 (85.7)	673 (95.1)	1026 (94.4)	
Non-permanent	14 (12.5)	36 (4.8)	24 (3.2)		12 (14.3)	35 (4.9)	61 (5.6)	
Employment at follow-up				<0.001				<0.001
Gainfully employed	41 (36.6)	742 (98.4)	758 (100)		41 (48.8)	686 (96.9)	1087 (100)	
Not gainfully employed	71 (63.4)	12 (1.6)	0 (0.0)		43 (51.2)	22 (3.1)	0 (0.0)	
Downsizing scale				<0.001				<0.001
Minor (less than 8 %)	30 (26.8)	394 (52.2)	n.a.		32 (38.1)	465 (65.7)	n.a.	
Larger (≥8 %)	82 (73.2)	360 (47.8)	n.a.		52 (61.9)	243 (34.3)	n.a.	
Past redundancies				<0.001				<0.001
Survived layoff or lost job	36 (32.1)	162 (21.5)	41 (5.4)		25 (29.8)	146 (20.6)	56 (5.1)	
No	76 (67.9)	592 (78.5)	717 (94.6)		59 (70.2)	562 (79.4)	1031 (94.9)	
Long-term sickness				<0.001				<0.001
Yes	5 (4.5)	2 (0.3)	0 (0.0)		4 (4.8)	6 (0.9)	2 (0.2)	
No	107 (95.5)	752 (99.7)	758 (100)		80 (95.2)	702 (99.1)	1085 (99.8)	
Depression at baseline				0.009				0.004
No depression (score <10)	82 (73.2)	616 (81.7)	649 (85.6)		54 (64.3)	527 (74.4)	856 (78.7)	
Depression symptoms (score 10–16)	23 (20.5)	115 (15.2)	91 (12.0)		19 (22.6)	135 (19.1)	177 (16.3)	
Major depression (score 17–24)	7 (6.3)	23 (3.1)	18 (2.4)		11 (13.1)	46 (6.5)	54 (5.0)	
Depression at follow-up				<0.001				<0.001
No depression (score <10)	83 (74.1)	633 (84.0)	676 (89.2)		54 (64.3)	526 (74.3)	880 (81.0)	
Depression symptoms (score 10–16)	18 (16.1)	99 (13.1)	64 (8.4)		20 (23.8)	129 (18.2)	152 (14.0)	
Major depression (score 17–24)	11 (9.8)	22 (2.9)	18 (2.4)		10 (11.9)	53 (7.5)	55 (5.0)	

Note: Values in the table are numbers (percentages) and means ± SD. SD = standard deviation. n.a. = not applicable

*p values from Pearson’s *χ*2 test for categorical variables or from analysis of variance for continuous variables

### Social causation: exposure to downsizing as a risk factor for subsequent depression

Table 2 shows the results of the multivariate multinomial logistic regression examining the associations between the exposure to downsizing during the Great Recession and depression at follow-up. We used workers with no depression at baseline and no downsizing as the reference group. Relative risk ratios are displayed according to the level of depression at baseline. In the total sample including men and women, we found significant effects of job displacement, with a more than threefold risk of incident major depression and twofold risk of less severe symptoms among displaced workers with no depression at baseline. The associations were weaker for survivors of layoffs with no depression at baseline; they were significantly more likely to suffer only from less severe incident symptoms (RRR = 1.35, *p* < 0.01). Gender-specific analyses indicate that job displacement was more detrimental in men than in women. A nearly fivefold risk of incident major depression was observed in unemployed men with no depression at baseline; the respective increase in unemployed women was about threefold, all *p* < 0.05. In contrast, surviving layoffs appeared to produce significant adverse effects in women (RRR = 1.59, *p* < 0.05 for incident major depression) but not in men (RRR = 1.05, *p* > 0.05).

**Table 2:** Results of the multinomial logistic regression models: effects of prior downsizing on depression

	Depressive symptoms at follow-up (score 10–16)			Major depression at follow-up (score 17–24)		
Both sexes (*N* = 3503)	No (Cases)	RRR (95 % CI)	*p* value	No (Cases)	RRR (95 % CI)	*p* value
Baseline depression scores 0–9 (no depression)						
Employees, no downsizing	1505 (115)	1 (ref.)		1505 (26)	1 (ref.)	
Downsizing survivors	1143 (114)	1.35 (1.08 to 1.69)	0.007	1143 (24)	1.36 (0.94 to 1.96)	0.105
Downsizing, displaced workers	136 (21)	2.01 (1.16 to 3.50)	0.013	136 (5)	3.66 (1.65 to 8.08)	0.001
Baseline depression scores 10–16 (depression symptoms)						
Employees, no downsizing	268 (79)	5.57 (4.44 to 7.00)	<0.001	268 (25)	8.43 (5.72 to 12.42)	<0.001
Downsizing survivors	250 (94)	7.54 (5.49 to 10.36)	<0.001	250 (27)	11.44 (6.71 to 19.51)	<0.001
Downsizing, displaced workers	42 (10)	11.23 (6.16 to 20.47)	<0.001	42 (10)	30.82 (12.67 to 74.98)	<0.001
Baseline depression scores 17–24 (major depression)						
Employees, no downsizing	72 (22)	7.86 (5.21 to 11.85)	<0.001	72 (22)	36.03 (22.35 to 58.08)	<0.001
Downsizing survivors	69 (20)	10.63 (6.66 to 16.97)	<0.001	69 (24)	48.91 (26.67 to 89.68)	<0.001
Downsizing, displaced workers	18 (7)	15.83 (7.94 to 31.55)	<0.001	18 (6)	131.731 (51.45 to 337.28)	<0.001
**Men** **(** ***N*** **= 1624)**						
Baseline depression scores 0–9 (no depression)						
Employees, no downsizing	649 (37)	1 (ref.)		649 (7)	1 (ref.)	
Downsizing survivors	616 (49)	1.42 (0.99 to 2.04)	0.058	616 (7)	1.05 (0.53 to 2.10)	0.890
Downsizing, displaced workers	82 (10)	1.64 (0.66 to 4.12)	0.290	82 (2)	4.93 (1.23 to 19.69)	0.024
Baseline depression scores 10–16 (depression symptoms)						
Employees, no downsizing	91 (25)	7.16 (5.00 to 10.26)	<0.001	91 (6)	11.46 (5.76 to 22.82)	<0.001
Downsizing survivors	115 (46)	10.17 (6.11 to 16.91)	<0.001	115 (8)	12.04 (4.62 to 31.39)	<0.001
Downsizing, displaced workers	23 (5)	11.76 (4.37 to 31.63)	<0.001	23 (8)	56.46 (11.52 to 276.58)	<0.001
Baseline depression scores 17–24 (major depression)						
Employees, no downsizing	18 (2)	4.36 (1.95 to 9.76)	<0.001	18 (5)	40.33 (16.44 to 98.92)	<0.001
Downsizing survivors	23 (4)	6.19 (2.56 to 14.95)	<0.001	23 (7)	42.35 (13.72 to 130.71)	<0.001
Downsizing, displaced workers	7 (3)	7.16 (2.10 to 24.39)	0.002	7 (1)	198.65 (34.61 to 1140.11)	<0.001
**Women** **(** ***N*** **= 1879)**						
Baseline depression scores 0–9 (no depression)						
Employees, no downsizing	856 (78)	1 (ref.)		856 (19)	1 (ref.)	
Downsizing survivors	527 (65)	1.32 (1.00 to 1.76)	0.052	527 (17)	1.59 (1.02 to 2.46)	0.039
Downsizing, displaced workers	54 (11)	2.12 (1.03 to 4.34)	0.041	54 (3)	3.17 (1.16 to 8.70)	0.025
Baseline depression scores 10–16 (depression symptoms)						
Employees, no downsizing	177 (54)	4.69 (3.49 to 6.29)	<0.001	177 (19)	7.02 (4.38 to 11.27)	<0.001
Downsizing survivors	135 (48)	6.20 (4.12 to 9.33)	<0.001	135 (19)	11.14 (5.82 to 21.30)	<0.001
Downsizing, displaced workers	19 (5)	9.92 (4.56 to 21.60)	<0.001	19 (2)	22.28 (7.29 to 68.13)	<0.001
Baseline depression scores 17–24 (major depression)						
Employees, no downsizing	54 (20)	9.90 (5.98 to 16.37)	<0.001	54 (17)	39.04 (21.81 to 69.89)	<0.001
Downsizing survivors	46 (16)	13.10 (7.33 to 23.40)	<0.001	46 (17)	61.91 (29.52 to 129.82)	<0.001
Downsizing, displaced workers	11 (4)	20.96 (8.75 to 50.22)	<0.001	11 (5)	123.85 (38.46 to 398.78)	<0.001

Note: Dependent variable: level of depression at follow-up. Reference outcome: no depression at follow-up (score 0–9). Analyses adjusted for: demographic (age, gender, education, marital status) and employment variables (permanence of employment as of 2008 and employment in 2010), exposure to past downsizing (2006–2008) and long term sickness. RRR by exposure status were computed as point estimates for linear combination of coefficients after multinomial logistic regression models

*RRR* relative risk ratios, *95 % CI* 95 % confidence interval, *No* number of persons, total; Cases: persons with the respective scores

### Health-related selection: pre-existing depression as a risk factor for subsequent job displacement due to organisational downsizing

Table 3 displays the results of the multivariate multinomial logistic regression estimating the risk of being laid off when organisations downsize in relation to pre-existing depression. Included were only persons who were displaced or survived layoffs during the Great Recession. We used workers who survived layoffs and remained employed in downsized organisations as the reference outcome. Relative risk ratios are shown according to the level of depression at baseline. In the analysis including both sexes, the likelihood of job displacement appears to grow with the increasing severity of depression: the adjusted relative risk ratios vary from 1.32 for less severe depressive symptoms (*p* > 0.05) to 1.93 for major depression (*p* < 0.05) compared with no depression. Gender-specific analyses indicate that men with major depression or less severe symptoms at baseline had no significant increase in risks of becoming unemployed subsequently. Estimates in women were, however, statistically significant for major depression (RRR = 2.18, *p* < 0.05).

**Table 3:** Results of the multinomial logistic regression models: effects of pre-existing depression on job displacement during downsizing

Both sexes (*N* = 1658)	No (job displacement)	RRR (95 % CI)	*p* value
Level of depression at baseline:			
No depression (score <10)	1279 (136)	1 (ref.)	
Depression symptoms (score 10–16)	292 (42)	1.32 (0.88 to 1.95)	0.183
Major depression (score 17–24)	87 (18)	1.93 (1.05 to 3.55)	0.035
**Men** **(** ***N*** **= 866)**			
Level of depression at baseline:			
No depression (score <10)	698 (82)	1 (ref.)	
Depression symptoms (score 10–16)	138 (23)	1.41 (0.83 to 2.42)	0.208
Major depression (score 17–24)	30 (7)	1.36 (0.47 to 3.93)	0.568
**Women (** ***N*** **= 792)**			
Level of depression at baseline:			
No depression (score <10)	581 (54)	1 (ref.)	
Depression symptoms (score 10–16)	154 (19)	1.23 (0.68 to 2.25)	0.495
Major depression (score 17–24)	57 (11)	2.18 (1.01 to 4.69)	0.046

Note: Analysis includes victims (i.e. displaced workers) and survivors of layoffs during the Great Recession. Dependent variable: unemployed through downsizing. Reference outcome: survivors of layoffs. Analyses adjusted for: demographic covariates (age, gender, education and marital status), permanence of employment as of 2008, exposure to past downsizing (2006–2008), downsizing scale and long term sickness

*RRR* relative risk ratios, *95 % CI* 95 % confidence interval, *No* number of persons with the respective scores; job displacement: unemployed through downsizing

## Discussion

### Key findings

The aim of this paper was to assess evidence of social causation and health selection in the context of organisational downsizing. From the raw data, we found that displaced workers and layoff survivors had higher prevalence of depression than workers not exposed to downsizing. The results of multivariate analyses suggest that job loss is a consistent predictor of major depression regardless of gender, with a somewhat greater effect size in men. At the same time, we found gendered effects of surviving a layoff: this exposure was significantly associated with subsequent major depression in women but not in men. Further results imply that health-related selection operates in women: pre-existing major depression increases the risk of being laid off when organisations downsize. However, we did not find strong evidence that depression influences the risk of job loss in men.

### Comparison with other studies and explanation of results

Since the earliest quantitative findings linking mental hospital admissions to employment loss during national recessions [42], it has been debated whether poor health results from or is caused by changes in employment status. Our finding that both processes may operate is in line with the very few prospective studies published so far [15–17, 43, 44]. We were able to add to this literature by analysing the gender-specific associations in the context of organisational downsizing. Previous research documented mostly detrimental health effects of downsizing [45, 46] but yielded inconclusive results on gender differences by measures of exposure. Our study concurs with other evidence that job loss raises risk of mental ill health in both genders [11, 47]. Several studies and meta-analyses found that men are more distressed by unemployment than women [47, 48], others showed worse mental health in unemployed women [49], whereas a third group of studies documented null overall findings in both genders combined [50]. An earlier analysis reported gender differences in the association between surviving a layoff and psychotropic drug prescription, with substantially larger effects in men [11]. By contrast, our analyses revealed that remaining employed in downsized organisations is much more detrimental for women. The mixed evidence can be attributed to methodological diversity of research, yet it is also important to acknowledge that gender differences in health effects might result from different gender regimes of paid and unpaid work in the family and society. Sweden represents a gender regime with a similar need for employment among men and women. With employment rates relatively close to that of men, women may experience similar psychosocial stress as men – and thus significant risks of depression – when unemployed. However, women are placed in a more precarious position than men with respect to their employment contracts. In balancing work and family life, women are more often found in temporary or part-time work with higher job insecurity [22, 47]. Women may therefore be more sensitive to the depressogenic effect of job insecurity when they survive layoffs. Furthermore, gender inequalities in employment and working conditions may intensify selection of women with mental ill health in unemployment when organisations downsize. In terms of health selection, our findings are consistent with the analyses suggesting an increased risk of exclusion from employment among women with mental ill health [44, 51]. We failed to replicate prior studies suggesting higher risks of transition to unemployment among men with mental health problems [51, 52].

It is important to interpret our results in the broader macroeconomic context of the global recession. Our findings differ from previous studies which were carried out in relatively stable times and reported no significant effects of downsizing survival [12] and only weak effects of health selection to unemployment in persons with mental ill health [48]. Downsizing may be especially distressing for the exposed workers when labour markets are weak, due to increased job insecurity, higher risks of further redundancies and bleak job opportunities [53]. It is also likely that such downsizing may intensify gender inequalities and social exclusion of women with major depression.

### Strengths and limitations

The methodological strengths include the unique population-based data collection approach and careful consideration of the downsizing exposures. Since prior depression is often the strongest predictor of subsequent ill health, we accounted for depressive symptoms at baseline when examining the later depressogenic effects of redundancies.

Nevertheless, some limitations need to be considered. First, although we included some register-based variables – age, sex and education, − the self-reported nature of other data poses concerns regarding response and common method biases [54]. We have therefore undertaken several steps to proactively address these issues. The questionnaire structure provided for a psychological separation of items dealing with depression and downsizing: queries on downsizing were placed in a final part of the large multi-topical questionnaire and included a cover sentence to make it appear that health measures are unrelated to downsizing. Temporal separation was ensured when data on our predictor and criterion variables were collected in two different SLOSH waves. By assessing exposure status through multiple questions, we could reduce the potential for acquiescence and social desirability. Finally, by assuring total anonymity and relying on voluntary participation, we could reduce the risks of common method bias. Though depressive symptoms were not validated by a psychiatrist in our study, we used the SCL-CD_6_ scale found earlier to be psychometrically valid for measuring depression severity in epidemiological research; its predictive validity has also been confirmed in relation to subsequent hospitalisations for depressive episodes and purchases of antidepressants [31]. However, symptoms of major depression assessed with the SCL-CD_6_ scale do not necessarily reflect the presence of a clinically significant disease; we did not collect data on depression treatment. Similarly, less severe symptoms do not necessarily imply a diagnosis of minor depression. Our results should be understood in relation to increased risk of depressive morbidity at clinical or mild subclinical levels.

Second, the attrition between the SLOSH waves may have biased the results because of selective responses. However, we found no significant impact of depression on the dropout; the analytic sample is representative for the sample of all respondents to the 2008 SLOSH wave with respect to depression prevalence. Therefore, we concluded that no serious selection problems occurred.

Third, we did not control for the possible confounding effects of, for instance, income and occupation grades. Instead, we used education as proxy for the socioeconomic status. Therefore, some residual confounding could have affected our results.

Finally, although we used data from a nationally representative Swedish workforce cohort, the displaced workers and layoff survivors do not constitute a random sample of all employees exposed to collective compulsory redundancies. Beyond the scope of this study were workers who withdrew from the downsizing situations – those who quit, retired or found another job before becoming unemployed. Future research should investigate health effects in these groups and examine the variety of reasons for choosing to leave a job in the downsized company.

Our findings should be generalizable to labour market participants in various occupations, both permanent and temporary workers in Sweden, who were in the scope of this study and could be affected by personnel downsizing to a different extent. The findings are not necessarily generalizable to other countries with different gender regimes and less extensive systems of social protection. Further research in a broader range of countries is therefore needed.

## Conclusions

The evidence from this study suggests that the relative importance of social causation and health selection varies by gender in the context of organisational downsizing. While job loss is important for inequalities in depression in both sexes, surviving layoffs is detrimental for women only. In addition, women with major depression have increased risks of exclusion from employment when organisations downsize. Given that downsizing is common in business life, our findings highlight the need for employment and mental health policies to successfully address morbidity related to threatening or implemented layoffs. Employers and health professionals should pay attention to health conditions among employees during layoffs. This seems to be of particular relevance for women. Gender specific practices and policies preventing social exclusion can be important to support female workers at higher risk of depression during layoffs.
